# Therapeutic strategies for autism: targeting three levels of the central dogma of molecular biology

**DOI:** 10.1038/s41398-023-02356-y

**Published:** 2023-02-16

**Authors:** Derek Hong, Lilia M. Iakoucheva

**Affiliations:** 1 Department of Psychiatry, University of California San Diego, La Jolla, CA USA; 2 Division of Biological Sciences, University of California San Diego, La Jolla, CA USA; 3 Institute for Genomic Medicine, University of California San Diego, La Jolla, CA USA

**Keywords:** Molecular neuroscience, Personalized medicine, Autism spectrum disorders

## Abstract

The past decade has yielded much success in the identification of risk genes for Autism Spectrum Disorder (ASD), with many studies implicating loss-of-function (LoF) mutations within these genes. Despite this, no significant clinical advances have been made so far in the development of therapeutics for ASD. Given the role of LoF mutations in ASD etiology, many of the therapeutics in development are designed to rescue the haploinsufficient effect of genes at the transcriptional, translational, and protein levels. This review will discuss the various therapeutic techniques being developed from each level of the central dogma with examples including: CRISPR activation (CRISPRa) and gene replacement at the DNA level, antisense oligonucleotides (ASOs) at the mRNA level, and small-molecule drugs at the protein level, followed by a review of current delivery methods for these therapeutics. Since central nervous system (CNS) penetrance is of utmost importance for ASD therapeutics, it is especially necessary to evaluate delivery methods that have higher efficiency in crossing the blood-brain barrier (BBB).

## Introduction

Autism Spectrum Disorder (ASD) is a neurodevelopmental disorder (NDD) that is characterized by three core symptoms: the deficits in social interaction and communication, language development, and restrictive and repetitive behaviors. A large proportion of children diagnosed with ASD manifest additional symptoms including cognitive deficits, developmental delay, anxiety and other medical comorbidities with mood and psychiatric disorders [1, 2]. As of 2021, the CDC has approximated that 1 in 44, or ~2.3%, of children in the United States have ASD diagnosis [3].

The estimates suggest that 70% of individuals with ASD have limited ability to live independently [4]. This lifelong dependency on caregivers as well as ASD-associated social, cognitive, and behavioral deficits can contribute to parental stress, leading to increased divorce rates in parents of ASD-diagnosed children [5]. At a financial level, the projected cost of the resources needed to care for individuals with ASD will progressively increase to $5.54 trillion/year by 2060 due special education costs, productivity loss due to informal caretaking, and increased use of healthcare services [6]. Given the prevalence of diagnosis, familial stress, and societal financial burden, it is imperative to develop and refine techniques to alleviate the social, cognitive, and behavioral symptoms in ASD.

It is well-established that ASD has strong genetic basis. Earlier studies demonstrated that monozygotic twins have significantly greater concordance for ASD than dizygotic twins, and ASD heritability is estimated to be 83% [7]. While individuals with some monogenic causes, such as Angelman Syndrome (AS), Fragile X Syndrome (FXS), and Rett Syndrome (RTT) [8–10], have features of ASD, the etiology of ASD as a whole is extremely heterogeneous [11, 12]. Earlier studies have identified rare de novo and inherited copy number variants (CNV) as major contributors to the increased risk for ASD [13–19]. Subsequently, whole exome sequencing (WES) of simplex families with one affected child demonstrated strong association of rare de novo exonic single nucleotide variants (SNV) with ASD [20–24], with more recent analyses highlighting around a hundred genome-wide significant ASD risk genes [25, 26]. For a subset of genes that are highly past genome-wide significant cut-off (at least FDR < 0.05), such as *KMT2E*, *ANKRD11*, *ARID1B*, *CHD8*, *PTEN, SHANK3, DYRK1A*, and *CUL3*, mouse models have been developed over the years [27–34]. Furthermore, non-human primate (NHP) models have been established for the *MECP2* (implicated in RTT) and *SHANK3* genes within cynomolgus macaques (*Macaca fascicularis*) to better recapitulate human developmental time points [35, 36]. These models have further implicated heterozygous loss-of-function (LoF) mutations (also known as haploinsufficiency) in these genes as responsible for specific neurobiological and behavioral animal phenotypes. In addition to rare de novo variants, a recent genome-wide association study (GWAS) has identified 5 genome-wide-significant ASD loci [37]. Given such an extreme genetic heterogeneity, and an unequivocal role of LoF-impacted (and often haploinsufficient) genes in ASD etiology, it will be invaluable to shift from identification-based research and proceed to investigate therapeutic techniques that could increase the expression of these genes.

**Fig. 1:**
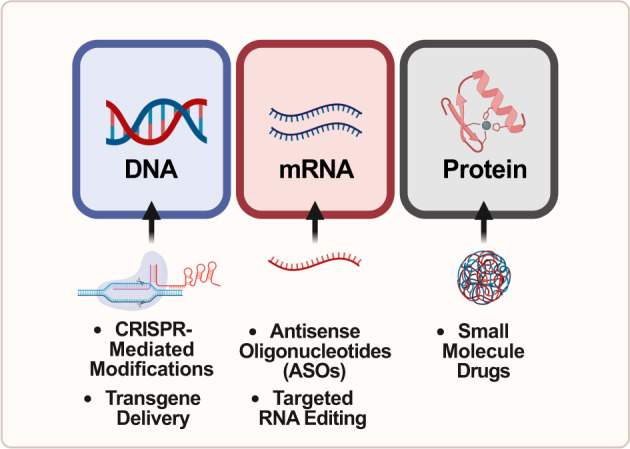
Overview of therapeutics for ASD at different levels of the Central Dogma of Molecular Biology that were discussed in this review. The figure lists therapeutics linearly based on the natural progression of gene expression. In the first column, small molecule drugs and CRISPR-based therapeutics are usually developed to treat diseases at the DNA level. In the second column, antisense oligonucleotides (ASOs) are usually developed to treat diseases at the mRNA level. In the third column, small molecule drugs are usually developed to treat diseases at the protein level. Specific examples of each of these therapeutics are described throughout the review.

The therapeutic interventions in ASD aimed at rescuing haploinsufficiency of individual genes could be developed to target all three levels of the central dogma of molecular biology: DNA, mRNA, and protein **(**Fig. 1**)**. Examples of such interventions include genome editing with CRISPR at the genetic level, antisense oligonucleotides (ASOs) at both, the transcriptional and post-transcriptional level, and the use of small-molecule drugs to target molecular pathways at the translational, or protein level. This review will analyze the advantages and disadvantages of the various techniques across the central dogma in order to rescue ASD-associated phenotypes.

## Rescue at the DNA Level

Gene therapy encompasses techniques that can alter the expression of an organism’s genes by targeting the DNA either through transgene delivery, or by direct modification of the genome, with the goal of therapeutically restoring a pathologically expressed gene to normal expression levels [38].

### Transgene delivery

The delivery of a transgene is a method of gene therapy that could be used to correct haploinsufficiency caused by LoF mutations [39]. It has been applied to several NDDs, for which monogenic cause is known. RTT is an NDD that lies within the classification of ASD [40, 41]. While the LoF of the Methyl-CpG-binding Protein 2 (*MECP2*) gene is causative to RTT, a duplication of the *MECP2* results in *MECP2* Duplication Syndrome (MDS) [10, 42]. It has been demonstrated that it is possible to reduce the severity of RTT through the delivery of the *Mecp2* transgene within a Mecp2-null mouse model [43]. However, given dosage sensitivity of the *MECP2* gene, proper dose determination is needed prior to clinical translation. A more recent study demonstrated that the delivery of instability-prone *Mecp2* (*iMecp2*) transgene cassette using an adeno-associated virus (AAV) vector in symptomatic *Mecp2* mutant mice significantly improved locomotor activity, lifespan and normalized gene expression [44].

Fragile X Syndrome, another NDD that is characterized by intellectual disability (ID) among other symptoms, has high comorbidity with ASD and is a result of a CGG triplet repeat expansion mutation in the fragile X mental retardation 1 gene (*FMR1*) that silences the production of its encoded FMRP protein [45, 46]. One study utilized transgene delivery of the unexpanded copy of the *FMR1* gene using an AAV vector directly injected into the brains of the Fmr1^-/-^ mice, and this successfully rescued repetitive behavioral, social and seizure mouse phenotypes [47]. Outside the context of NDDs, the FDA has approved Luxturna—a transgene therapy to deliver the *RPE65* gene to the retina and effectively treat a rare inherited retinal disease that results in vision impairment and blindness [48, 49]. Given the approval of Luxturna and successes in transgene delivery for the RTT and FXS mouse models, this mechanism may be of value for other LoF genes implicated in ASD. However, it is important to note that NDD therapeutics may face more obstacles in clinic than the retinal delivery of Luxturna as the brain is less accessible than the eye and requires the delivery vector to cross the blood-brain barrier (BBB). Furthermore, the brain is a more complex organ with greater complexity in cell types and significantly varying levels of gene expression between each type, making cell-specificity an additional concern in NDD therapeutics [50].

### CRISPR-mediated modifications

Within the past decade, the advent of genome engineering with CRISPR-Cas9 has revolutionized gene therapy, opening a new therapeutic avenue based on DNA-level modifications. CRISPR-Cas9 is a construct consisting of a guide RNA (gRNA) that targets the genetic loci of interest and the Cas9 endonuclease enzyme, which functions as a pair of nucleotide scissors that cleave the DNA at the target site—effectively generating a double-stranded DNA break for subsequent genome editing [51]. The unique ability of targeting Cas9 to any location in the genome has opened new avenues for targeted gene therapy.

One possibility of harnessing genome engineering for regulating gene expression is to direct it towards natural antisense transcripts (NATs) [52]. NATs are endogenously expressed in both prokaryotic, and eukaryotic organisms. In eukaryotic systems, NATs can have a bidirectional regulatory effect on the transcription of their target genes, either suppressing or enhancing the translation of the target gene’s mRNA [53, 54]. Inhibition of the target gene expression can occur through various mechanisms such as RNA interference, once the sense-antisense mRNA duplex has formed, transcriptional interference, in which the NAT can act as a physical barrier for RNA polymerase activity, or epigenetic methylation of the sense gene DNA, thus inhibiting the transcription of the sense mRNA transcript [55, 56]. Although many NATs have inhibitory control over the expression of their complimentary genes, there are some cases in which NATs can directly increase sense gene expression [57]. With enhancement of target gene expression, it is proposed that NATs can increase the expression of the target gene through increasing the stability of the sense mRNA or euchromatin-associated epigenetic modifications [54]. Putting this into a therapeutic context, it may be possible to restore the expression of ASD risk genes that have LoF mutations through targeted suppression of the respective inhibitory NATs.

This strategy has recently been applied to Angelman Syndrome (AS), an NDD that can be driven by a LoF mutation in the maternal copy of the *UBE3A* allele [58, 59]. Since the paternal copy of *UBE3A* is normally inactive, there is therapeutic value in investigating the inhibition of the *UBE3A* NAT. There has been success in rescuing haploinsufficiency of the *UBE3A* gene through CRISPR-Cas9-mediated transcriptional inhibition of the *UBE3A* NAT in mice—effectively restoring *UBE3A* expression through re-activation of the paternal copy [58, 60]. In the context of FXS, instead of targeting NATs of the *FMR1* gene, there has been success in restoring *FMR1* expression in induced pluripotent stem cells (iPSCs) through direct CRISPR-Cas9-mediated deletion of pathological repeat sequences of the sense gene [61].

Outside the context of NDDs, there are currently attempts of using CRISPR-Cas9 to treat Leber congenital amaurosis 10 (LCA10). LCA10 is a severe form of retinal dystrophy caused by an adenine to guanine point mutation within intron 26 of the human CEP290 gene (IVS26)—resulting in the inclusion of a cryptic exon and a premature stop codon [62]. To target LCA10, one study developed a therapeutic called EDIT-101 that consists of Cas9 and a gRNA construct packaged into AAV serotype 5 (AAV5) to introduce a deletion or inversion within the mutated region of intron 26 [63]. In a human CEP290 IVS26 knock-in mouse model, EDIT-101 had strong productive editing rates within photoreceptor cells, effectively restored normal splicing of the CEP290 mRNA, and restored the production of the full-length CEP290 protein [63]. At the clinical level, EDIT-101 is the first use of a genome editor in the CNS and is currently in phase 1/2 clinical trials in adult and pediatric patients (https://clinicaltrials.gov/ct2/show/NCT03872479).

### Modified CRISPR activation

**Fig. 2:**
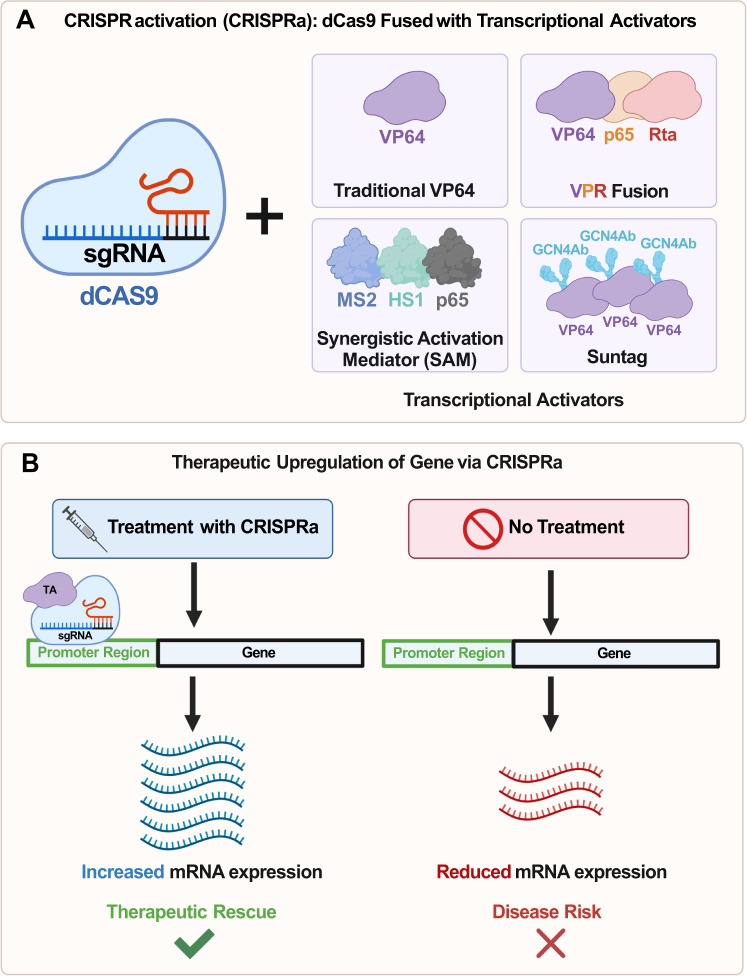
Schematics of CRISPR activation (CRISPRa) and its’ role in ASD therapeutics. CRISPRa uses a catalytically dead Cas9 (dCas9) fused with various transcriptional activators. **A** Depiction of the most common transcription activators (TA) fused to dCas9: (i) The traditional VP64, (ii) A combination of VP64, p65, and Rta (VPR), (iii) The synergistic activation mediator (SAM) system consisting of p65, HSF1, and MS2, and (iv) The Suntag system consisting of dCas9 fused to a polypeptide chain in which a VP64-GCN4 antibody fusion is bound to each peptide—allowing for multiple copies of VP64 to be recruited. **B** Mechanism of action for CRISPRa. When guided to the promoter region, 1 to 1000 bp upstream of the transcription start site (TSS) of the target gene by a sgRNA construct, the dCas9-VPR will promote an increase in transcription. The reduction in mRNA expression on the right column is a result of the untreated Haploinsufficient gene. *Abbreviations*: TA Transcriptional Activator, Rta Epstein-Barr Virus R Transactivator, MS2 - Bacteriophage Coat Protein, HSF1 Human Heat-Shock Factor 1, GCN4Ab GCN4 Antibody.

Using the traditional CRISPR-Cas9 complex as a basis, a modified version in which the Cas9 enzyme is inactive or “dead” (dCas9) has been developed [64]. While the endonuclease-mediated cutting ability in dCas9 is rendered inactive, the enzyme can still bind—allowing for specific targeting within the genome. This dCas9 system can be fused with activators of transcription (Fig. 2A) in order to increase the expression of genes without inducing a double-stranded DNA break—a process known as CRISPR-mediated activation (CRISPRa) [65–67] (Fig. 2B). For CRISPRa to work, single-guide RNAs (sgRNA) are designed to target regions between 1 to 1000 bp upstream of the transcription start site (TSS) within the promoter region of the target gene. Once the sgRNA has bound to this region, the dCas9 fused to the appropriate transcriptional activators will be recruited.

It is possible to modulate the magnitude of activation by changing which of the proteins are fused to the dCas9 (Fig. 2A). When fused to C-terminus of dCas9, the VP64 transcriptional activator produces modest increases in gene expression [68]. Building upon this initial VP64 fusion, the combinatory fusion of VP64, p65, and Rta (VPR) to the C-terminus of dCas9 produces significantly higher transcriptional activation, with an effect size ranging from 22-fold to 320-fold greater than VP64 alone [68]. Tangentially, SunTag is another system that makes use of VP64. However, instead of having VP64 directly fused to dCas9, Suntag consists of dCas9 fused to a polypeptide chain in which a VP64-GCN4 antibody fusion is bound to each peptide—allowing for multiple copies of VP64 to be recruited to a gene’s promoter [69]. Another dCas9 fusion is the synergistic activation mediator (SAM) system, which consists of three total components: dCas9 fused to VP64 with a nuclear localization sequence (NLS) (NLS-dCas9-VP64), two activation domains (p65 and human heat-shock factor 1 (HSF1)) fused to the MS2 bacteriophage coat protein (MS2-p65-HSF1), and the sgRNA fused to two MS2 protein-targeting hairpin aptamers at the stem-loop 2 and tetraloop regions (sgRNA2.0) [70].

Comparatively, one study demonstrated that while VPR, Suntag, and SAM systems had multiple orders of magnitude greater transcriptional activation than VP64 alone, there was no more than a single level of magnitude in differences between these three systems [71]. Though it is important to note that the differences between VPR, Suntag, and SAM are cell-type and gene-specific. Furthermore, it may not be advantageous to ubiquitously use a potent transcriptional activator in CRISPRa as over-activation of some genes may induce unwanted effects. For example, in case of *MECP2*, it may be preferable to induce moderate activation through the traditional dCas9-VP64 instead of the more robust activators as over-activation of *MECP2* could potentially result in MDS. This CRISPRa system that has been described has recently been applied in several animal models of NDDs.

Dravet Syndrome is an NDD caused by haploinsufficiency of the *SCN1A* voltage-gated Na^2+^ channel [72, 73]. Through targeting long non-coding RNA [74] or the promoter region of the *SCN1A* gene with CRISPRa [75], the studies successfully increased *SCN1A* expression and restored dysfunctional neuron excitability and seizure phenotypes. Similarly, haploinsufficiency of the *SCN2A* voltage-gated Na^2+^ channel has also been implicated in ASD [76, 77]. One recent study used CRISPRa to successfully restore neuronal excitability and electrophysiological deficits in *Scn2a*^+/-^ mice to wild-type levels, an effect that persisted until at least 3 and 8 months, respectively [78].

Outside the context of NDDs, CRISPRa has been used to target the *KCNA1* voltage-gated potassium channel to rescue seizure frequency and cognitive dysfunctions in a mouse model of epilepsy [79]. At the physiological phenotype level, CRISPRa has also been used to increase the transcription of obesity risk genes *SIM1* and *MC4R*, and to successfully rescue haploinsufficient obesity in mouse models—with the effects persisting up to 9 months post-treatment [80]. At the expression level, another study found that CRISPRa was successful in upregulating the expression of *TTR* within dCas9-SAM transgenic mice 19 days after treatment, but this effect diminished over the course of 8 months [81]. With the exception of these studies, there is still insufficient information about the extent to which the effects of CRISPRa therapy will persist. Successful clinical translation necessitates further in vivo studies that assess significantly later time points.

## Rescue at the mRNA level

Targeted inhibition of inhibitory NATs or activation of a gene using CRISPRa are examples of therapeutic strategies for NDDs at the DNA level. However, it is also possible to regulate the expression of NATs at the post-transcriptional level. When exploring the next level of regulation, it may be of value to assess the potential of using ASOs, as they can increase the expression of genes through various mechanisms. These mechanisms can be classified under two main functional categories: (a) upregulation of the sense gene through direct interactions with the sense gene mRNA transcript, and (b) upregulation of the sense gene through ASO-mediated inhibition of the NAT.

### Direct upregulation of sense gene

**Fig. 3:**
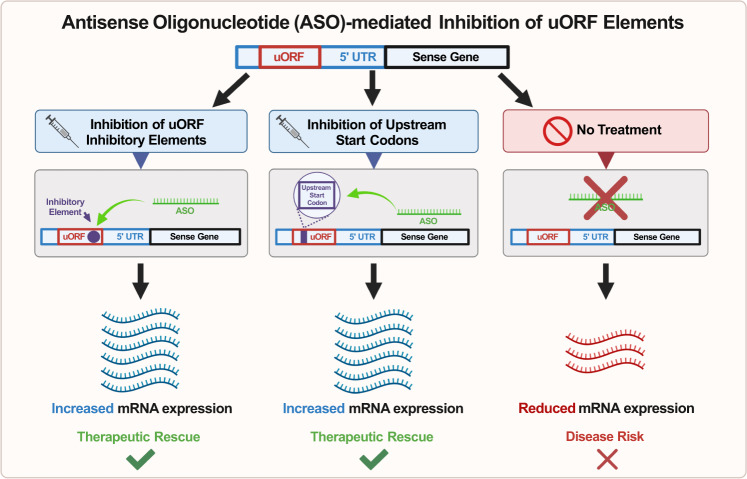
Diagram of inhibitory antisense oligonucleotide (ASO) mechanisms. **A** ASOs can be used in the inhibition of the upstream start codon within the upstream open reading frame (uORF) of an mRNA transcript to increase translational expression of the target gene; **B** ASOs can be used to target and hybridize with inhibitory elements that form translation-inhibiting secondary structures within the uORF of an mRNA transcript to ultimately increase translational expression of the target gene. The reduction in mRNA expression on the far-right column is a result of the untreated haploinsufficient gene.

When investigating the ability of ASOs to upregulate the sense gene through directly interacting with the sense gene mRNA, the first mechanism lies in ASOs that target the upstream open reading frames (uORFs) of the sense transcript [82] **(**Fig. 3**)**. The uORF is a region in the 5’ untranslated region (UTR) of the mRNA transcript that often contains an additional start codon, as well as a stop codon [83]. When translation is initiated at this loci, there can be a decrease in the efficiency of protein translation at the upstream start site due to the production of a peptide that ultimately blocks ribosomal function [84, 85]. To harness this mechanism of gene upregulation, ASOs were designed to specifically target the uORF of the *Lrpprc* gene in mouse models and found that the treatment was successful in increasing the LRPPRC protein expression, an experiment that provided evidence to the efficacy of this mechanism [84]. However, further studies are needed to investigate the success of uORF targeting in disease models as many of these studies have been proof of principle rather than demonstrating success in disease models.

Second, instead of mediating the cutting of mRNA transcripts, ASOs can also be designed to target and hybridize with inhibitory elements in the 5’ UTR region of the mRNA transcript. The presence of translation-inhibiting secondary structures within the 5’ UTR of mRNA transcripts has been previously demonstrated [82, 86], and designing ASOs that are complementary to these regions, could potentially relieve translational inhibition (Fig. 3).

For example, one study used an ASO to target a hairpin structure in the 5’ UTR region of the *LDLR* mRNA that was inhibitory to protein translation, which resulted in an increase of LDLR protein expression and LDL uptake in HEK293T cells [82]. Similar methods were used for cystic fibrosis (CF), a disease characterized by significant pulmonary and pancreatic dysfunctions and is a result of mutations in the *CFTR* gene, coding for a Cl^-^ channel [87]. In a cellular model of CF, ASOs were designed to target the inhibitory secondary mRNA structures in the uORF of the 5’ UTR on the *CFTR* mRNA transcript—effectively increasing both the expression and function of CFTR [88]. However, although success within the CF in vitro model provides potential for clinical translation, further evidence of in vivo success using this specific therapy approach is needed.

**Fig. 4:**
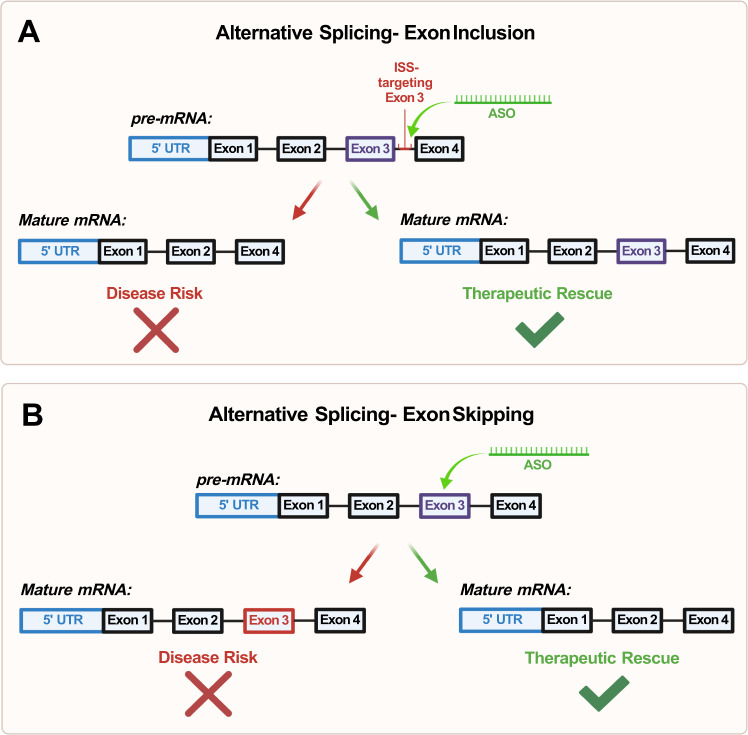
Diagram of alternative splicing regulation mechanisms by antisense oligonucleotides (ASOs). **A** Exon inclusion functions through targeting splice junctions and *cis*-regulatory elements (CRE) as a form of alternative splicing of an mRNA transcript, thus producing a functional copy of the protein. In this example, the ASO targets an intronic splicing silencer (ISS); **B** Exon skipping is a form of alternative splicing regulation that functions by masking the dysfunctional exon with the ASO—resulting in that exon being spliced out of the final mRNA transcript. This allows for the restoration of the reading frame and translation of a partially functional protein.

Third, there is evidence of successful restoration of aberrant mRNA splicing in non-NDD muscular conditions such as Spinal Muscular Atrophy (SMA) [89] and Duchenne Muscular Dystrophy (DMD) [90] through targeting splice junctions and cis-regulatory elements with ASOs [91, 92] (Fig. 4A). In SMA, individuals lack a working copy of the *SMN1* gene, and the ASOs are used to facilitate proper splicing of the *SMN2* gene through inducing the inclusion of exon 7—ultimately rescuing the expression of the SMN protein within in vitro mammalian cell models [91]

In 2016, the FDA authorized the use of Spinraza, the first drug-based therapy for SMA [93]. Spinraza functions through this mechanism of targeted exon 7 inclusion in the SMN2 mRNA, effectively rescuing gross motor functions in patients [94, 95]. In DMD, mutations in the dystrophin gene induce a frameshift and end up producing a non-functional dystrophin protein [96]. DMD-targeting ASOs are targeted towards exons that carry frameshift mutations responsible for DMD. The designed ASOs mask the dysfunctional exon in the pre-mRNA, resulting in the exon being spliced out of the final mRNA transcript—thus restoring the reading frame and producing a partially functioning copy of the dystrophin protein [92, 97] (Fig. 4B).

Although much promise was seen in early stages of clinical trials with the exon-skipping ASO Drisapersen for DMD, phase 3 trials failed to achieve clinical success [98–100]. However, in 2016 the FDA authorized the use of Eteplirsen, an ASO that induces exon-skipping to express partially functioning dystrophin, as the first drug-based therapy for DMD [101]. Similarly, an ASO promoting alternative splicing by the name of Milasen was designed as a personalized drug to treat an individual with Batten disease, a neurodegenerative disease characterized by blindness, an increased susceptibility to seizures, and developmental delay [102–104]. The mutation in the *MFSD8* (also known as *CLN7*) gene resulted in a truncated, and dysfunctional protein, and treatment with Milasen was able to effectively rescue seizure phenotypes and to increase neurological scores, temporarily improving quality of life of the patient [104].

**Fig. 5:**
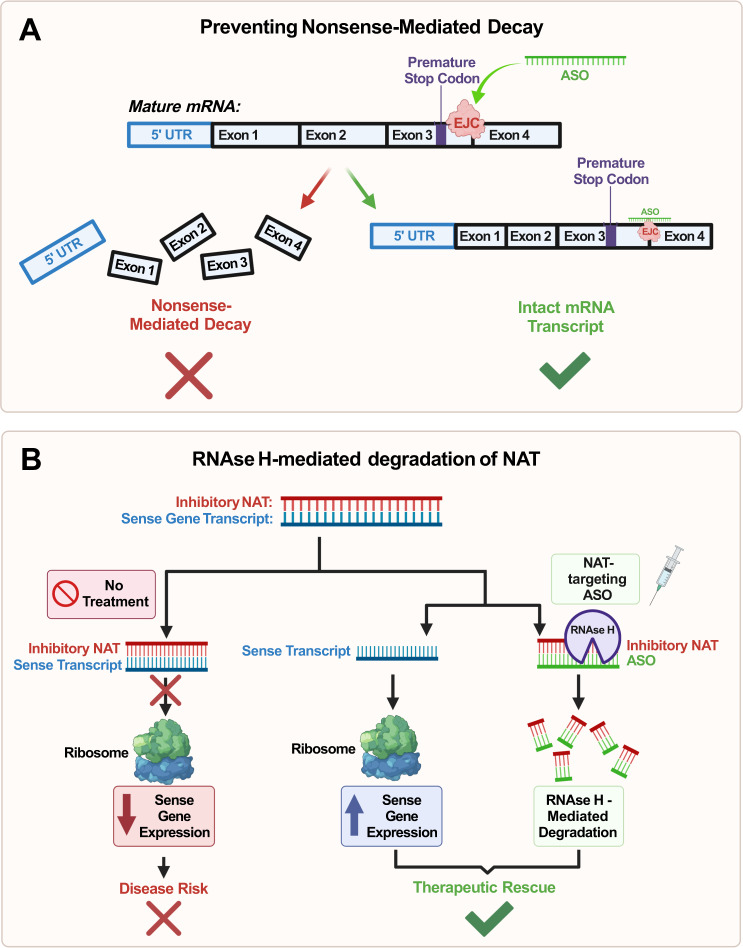
Diagram of antisense oligonucleotide (ASO) mechanisms pertaining to mRNA transcript degradation. **A** ASOs prevent the nonsense-mediated decay (NMD) of an mRNA transcript through hybridization. By targeting the exon-junction complex (EJC) region downstream of a transcript’s premature termination codons (PTC), the ASOs increase the stability of the transcript. This prevents NMD and increases the translational expression of the target gene; **B** ASOs can induce RNAse H-mediated degradation of a natural antisense transcript (NAT) to relieve inhibition of the sense mRNA transcript through hybridization. The RNAse H endonuclease enzyme will recognize and cleave the ASO-NAT duplex, thus allowing for unsuppressed translation of the respective sense gene. Paternal allele activation of UBE3A via a UBE3A-AS-Targeting ASO (most right column) is a specific example of this mechanism used to treat Angelman Syndrome.

Fourth, ASOs can suppress the nonsense-mediated decay (NMD) of mRNA transcripts by targeting the exon-junction complex (EJC) region located downstream of a transcript’s premature termination codons (Fig. 5A). Mechanistically, NMD is dependent on the presence of at least one EJC, and targeting this pathway with ASOs led to the increase of *MECP2* gene expression within in vitro mammalian cell models [105]. This provides evidence towards potentially using ASOs to inhibit NMD of *MECP2*, thus opening a therapeutic avenue for the treatment of Rett syndrome. However, it is again important to note *MECP2* dosage sensitivity as a significant roadblock to a successful treatment. Additionally, further studies are needed to determine the ideal dose to prevent induction of MDS, and more research is needed within in vivo models prior to successful clinical translation.

### Upregulation of sense gene via NAT degradation

While the previously mentioned mechanisms function to upregulate the sense gene by directly acting upon the sense gene mRNA transcript, one limitation lies in the fact that uORFs only exist in approximately 50% of mammalian mRNA and within this 50%, not all are inhibitory [106, 107]. Therefore, there is much value in the upregulation of ASD-associated sense genes through ASO-mediated NAT degradation. Mechanistically, once an ASO is bound to the NAT mRNA transcript, the RNAse H endonuclease enzyme will cleave the RNA duplex—effectively suppressing the inhibitory function of these NATs [59, 108] (Fig. 5B).

As previously discussed, Dravet Syndrome (DS) is an NDD caused by haploinsufficiency of the *SCN1A* voltage-gated Na^2+^ channel [72, 109]. With the use of the ASOs to target the *SCN1A* NAT, one study was effectively able to rescue the sense *SCN1A* gene expression, ameliorate neuronal excitability and seizures in a mouse model of DS—an effect that almost entirely recapitulates the rescued phenotypes observed via CRISPRa treatment [74, 75].

Likewise, Angelman Syndrome (AS) is an NDD driven by haploinsufficiency of the *UBE3A* gene. While previous success was found in CRISPR-based rescue at the DNA level, it is also possible to target the *UBE3A* NAT mRNA transcript for degradation at the transcriptional level [60]. Multiple studies have shown that it was possible to decrease the expression of the *UBE3A* NAT, rescue the sense *UBE3A* expression, and ameliorate cognitive and behavioral phenotypes in a mouse model of AS through the RNAse-mediated degradation [59, 110]. In support of this mechanism, the experimental drugs GTX-102 (https://clinicaltrials.gov/ct2/show/NCT04259281), ION582 (https://clinicaltrials.gov/ct2/show/NCT05127226), and RO7248824 (https://clinicaltrials.gov/ct2/show/NCT04428281) utilize NAT-targeting ASOs and are currently undergoing phase 1/2 clinical trials in pediatric and adult AS patients. However, it is important to note that the GTX-102 study was temporarily paused due to patients developing acute inflammatory polyradiculopathy as a result of the treatment [111]. It was reported that the polyradiculopathy was not an immune reaction, but likely the result of local toxicity from high concentration of GTX-102 at the site of injection [111]. Despite these adverse reactions, the therapeutic shows great promise as all participants demonstrated significant improvements in motor functions, communication, sleep, and behavioral issues.

### Targeted RNA-editing therapeutics

Outside of ASO-based therapeutics, which primarily function as post-transcriptional modulators, the future may hold promise in directly editing the mRNA transcript. With the help of Cas13, an RNA-guided ribonuclease traditionally used for effective and specific cleavage of mRNA transcripts, it was possible to develop a new fusion protein capable of editing the mRNA transcript known as RNA Editing for Programmable A to I Replacement (REPAIR) [112]. To develop REPAIR, the Cas13b ortholog was mutated to be catalytically dead Cas13b (dCas13b) and fused with the adenosine deaminase acting on RNA 2 (ADAR2) enzyme—allowing for the replacement of adenosine (A) residues to inosine (I) residues on mRNA transcripts. Similarly, fusion of the dCas9 enzyme to ADAR2 has been proven to perform the same A to I modifications in mRNA with comparable efficiency and specificity as the REPAIR system [113]. Building upon ADAR2-associated RNA editing, one recent study developed AAVs that co-express the wild-type human ADAR2 catalytic domain fused to bacteriophage λN peptide (Editase^wt^) and *Mecp2*-targeting guide RNAs [114]. The study used this Editase^wt^-based system to target *Mecp2*^*G311A*^ mutation, which creates a stop codon resulting in lack of MeCP2 protein. The retro-orbital injection of the construct into *Mecp2*^*G311A*^ mutant mice successfully restored MeCP2 protein expression and function, prolonged survival, and improved respiratory function of the mice. Although these ADAR-based, RNA editing therapeutics are highly specific to making A to I modifications in mRNA transcripts, they still provide a promising alternative therapeutic route to clinical translation of NDD therapeutics.

## Rescue at the protein level via small molecule drugs

Further downstream in the central dogma of biology, a key therapeutic approach lies in the activation or inhibition of molecular pathways implicated in NDDs. This would effectively shift focus from expression-based therapeutics to post-translational treatments.

### Proliferative pathways

Tuberous sclerosis (TS) is a monogenic disorder with a high prevalence of ASD that is caused by LoF mutations in the *TSC1* and *TSC2* genes [115]. These mutations lead to the hyperactivation of the mTOR pathway, ultimately resulting in the formation of benign tumors in numerous organs [116, 117]. Mouse studies have shown that mTOR inhibitors such as Rapamycin, which formerly received FDA approval for cancer treatments, successfully rescued deficiencies in social and repetitive behaviors in postnatal day 7 TSC-mutant mice, and only social deficits in 6-week-old TSC-mutant mice [118, 119]. However, translational attempts of mTOR inhibition have been unsuccessful in ameliorating cognitive functioning, behavioral problems, autism and neuropsychological deficits in children ages 4-17 treated with Everolimus—another mTOR inhibitor that was previously FDA approved for cancer treatments [120]. Given these results, it is important to consider the time point of therapeutic administration, as earlier intervention may yield greater treatment efficacy, as evidenced by the age-dependent success in the mouse studies.

### Rescuing inhibitory signaling pathways

It has been previously hypothesized that potential loss of balance in neuronal excitatory/inhibitory signals [121–123] within syndromic ASD subtypes such as FXS, RTT, AS, and in idiopathic ASD can be attributed to a decrease in the inhibitory GABA_A_ receptor function [124, 125]. Therefore, upregulation or gain in GABA_A_ receptor function could be a potential therapeutic target in rescuing ASD phenotypes. Arbaclofen, a GABA_B_ agonist, has recently emerged as a potential therapeutic for ASD. In a 16p11.2 deletion mouse model, Arbaclofen has been found to rescue memory deficits, measured by freezing behavior in context-dependent aversive learning tasks [126]. The same study found that Arbaclofen successfully rescued deficits in male-female social interactions, measured by nose-to-anogenital sniffing and time spent of the male following the estrous female. In a small clinical trial of 25 adolescents, Arbaclofen has been found to rescue slow auditory sensory processing in males with idiopathic ASD [127]. However, it is important to consider that while some success was found in an idiopathic ASD study, phase III clinical trials in child (ages 5-11), and adolescent through adult (ages 12-50) FXS patients proved to be unsuccessful as they were unable achieve the primary outcome of rescuing social deficits [128]. The results of this phase III clinical trial suggest that future trials should consider higher doses, larger sample sizes, a younger age group, and better outcome measures. Therefore, although stimulation of the GABA_B_ receptor using Arbaclofen to increase inhibitory neuronal signals and rescue the abnormal excitatory/inhibitory balance in ASD may show promise, the genetic heterogeneity of ASD may hamper its further use. Patient genetic stratification and better clinical outcome measures are clearly needed for future clinical trials in ASD.

### Rescuing excitatory signaling pathways

An alternative hypothesis is that an increase in the excitatory glutamate signaling can play a role in the dysregulation of neuronal excitatory/inhibitory signal balance. In FXS, it has been found that there was a causative increase in the metabotropic glutamate receptor 5 (mGluR5) that accompanies the loss of *FMR1* expression [129]. Molecular inhibition at the mGluR5 loci has been successful in multiple mouse models. In BTBR mice, the use of the mGluR5 antagonist MPEP successfully rescued the repetitive grooming phenotype [130]. MPEP also rescued not only the same repetitive grooming, but also anxiety-related marble-burying and locomotion in a valproic acid (VPA) mouse model [131]. Furthermore, treatment using the mGluR5 negative allosteric modulator (NAM) CTEP rescued the impaired memory in the 16p11.2 microdeletion mouse model [132]. Clinical translation, however, has been challenging, and preclinical trials for mGluR5 NAMs did not rescue phenotypes in human FXS patients [133]. Failures in the preclinical trials could possibly be attributed to improper dose extrapolation from mouse models, duration of treatments being too short, or the fact that mGluR5 inhibition is preferentially effective in a younger population—parameters that can be adjusted in future trials [134, 135]. Early continuous inhibition of group 1 mGlu signaling partially rescued dendritic spine abnormalities in the *Fmr1* knockout mouse model for fragile X syndrome [133].

Another recently discovered therapeutic strategy in ASD is targeting a small GTPase, RhoA, that is involved in cellular cytoskeleton structure and motility [136], and has found to be upregulated in some ASD models while being downregulated in others [34, 137–139]. Cullin3 (*Cul3*) is a genome-wide significant ASD risk gene [26], whose haploinsufficiency contributes to a decreased neuronal dendritic growth and a decreased neuronal network activity [34]. Since a *Cul3* haploinsufficient mouse model also showed an upregulation of RhoA expression, treatment using the RhoA inhibitor Rhosin was used to successfully rescue these phenotypes in vitro in primary cortical neuron cultures derived from these mice [34]. In a social defeat mouse model of affective stress, Rhosin has also been found to rescue behavioral phenotypes, such as impaired novel mouse interactions and avoidance behavior through suppression of the dopamine 1 receptor of medium spiny neurons (D1-MSNs) within the Nucleus Accumbens (NAc) [140]. Similarly, in *Kctd13* (a gene within 16p11.2 CNV) haploinsufficient and knockout mice, there was an increase in RhoA expression coupled with deficiencies in synaptic signaling—an effect that was ameliorated with Rhosin treatment [137]. These successes within mouse models provide evidence that Rhosin may be a valid therapeutic avenue for individuals with a deletion in the 16p11.2 or *Cul3* loci.

## Delivery of therapeutics

Viral delivery of CRISPR-Cas9 gene constructs and ASOs have been a commonly used method that utilizes vectors such as lentiviruses, adenoviruses, and adeno-associated viruses (AAV) [141] (Table 1). Though widely used, it is important to consider the potential drawbacks such as a person’s immunological response to these vectors, as well as limitations in packaging sizes. Although an adenovirus vector may have a larger packaging limit of up to ~36 kb, there is a greater risk of an inflammatory immunological response [142]. Conversely, AAVs have a limited packaging size of ~5 kb with significantly milder inflammatory risk [143]. Another important viral vector is lentivirus, which is a type of retrovirus that have a packaging size of ~9 kb—an intermediate between that of the adenovirus and AAV vectors [144]. One of the main advantages of lentivirus is its ability to deliver transgenes and integrate them into the genome for longer-lasting expression. However, this advantage contributes to the risk of off-target effects due to insertional mutagenesis, since lentiviruses do not have high specificity. Insertional mutagenesis is a result of the exogenous lentiviral DNA being integrated into the host genome at open regions and if this occurs at an off-target site, it could result in aberrant gene expression [145]. Studies have also shown that the lentivirus vector confers moderate inflammatory risk, but further studies in immunogenicity and prevention of recombination events are needed for optimized clinical translation [146].

**Table 1:** Comparison of viral vectors discussed in the review.

Viral vector	Packaging size (kb)	Immunogenicity	Genome integration	Blood-brain barrier (BBB) penetrance
Adenovirus	~36 kb	High Inflammatory Risk	No	No
Lentivirus	~9 kb	Moderate Inflammatory Risk	Yes (Insertional Mutagenesis)	No
Adeno-Associated Virus (AAV)	~4.5-5 kb	Mild Inflammatory Risk	Yes (Insertional Mutagenesis)	Yes • AAV9 • rAAV-PHP.B/eB (Mice) • AAV.CPP16 (Mice/NHP)

*NHP -* Non-Human Primates.

Despite earlier beliefs of AAVs being free of insertional mutagenesis, some studies suggest that AAV has a propensity to integrate randomly into the genome at double strand breaks (DSBs) [147], leading to tumorigenesis and liver cell hyperplasia in animals [148]. For example, in a mouse model of mucopolysaccharidosis (MPS VII), transgene delivery of b-glucuronidase in neonatal mice using an AAV2 vector resulted in the mice developing hepatocellular carcinoma (HCC)—with evidence of AAV integration within the tumors [149, 150]. Most of the AAV integrations occurred in the RNA imprinted and accumulated in nucleus (*Rian)* locus, which contributes to the epithelial to mesenchymal transition in cancer [148]. These studies also suggest that age plays a role in AAV-induced HCC development as *Rian* is expressed at a greater amount earlier in life. In a hemophilia dog model, there was evidence of AAV integration four years after being treated with canine factor VIII (cFVIII) in an AAV8/9 vector, however, there was no evidence of tumor formation within these dogs that had AAV integration [151].

The integrative propensity of AAVs is particularly important to consider in light of the genome editing therapies. Since AAVs have been found to integrate within DSBs with high frequency [152], the coupling of CRISPR-Cas9 with AAVs as the delivery vector must be carefully evaluated before clinical translation [58, 153]. Although no confirmed genotoxic events in humans have been reported from the use of rAAV vectors to-date, given the above evidence, it will be imperative to perform further studies in larger model organisms (such as NHPs) with longer time points. In addition to this, persistent monitoring and testing for HCC biomarkers will be necessary to perform in a clinical setting.

Many viral vectors do not penetrate the BBB with high efficiency and must be introduced via invasive direct injection or potentially neurotoxic disruption of the BBB [154]. Since most of the NDD risk genes are expressed in the brain, the sub-optimal delivery methods of these viral vectors could appear ill-suited for clinical delivery of NDD therapeutics. Although some AAV serotypes such as AAV1, AAV2, AAV5, and AAV8 can transduce neurons, they are unable to effectively cross the BBB through non-invasive intravenous injection [155]. The AAV9 serotype, however, has been found to have the ability to efficiently cross the BBB when intravenously delivered to neonatal and adult mice [156]. Therefore, unlike the adenovirus, lentivirus, and other AAV serotypes, the AAV9 vector is able to effectively avoid two main issues: the need for invasive injection, and compromising the integrity of the BBB, although its transduction efficiency diminishes with increasing age [156]. In an RTT mouse model, it was found that AAV9-mediated intracranial delivery of the MECP2 transgene effectively increased survival and some behavioral phenotypes [43]. In a phase 3 clinical trial, this AAV9 vector has found success in intravenously delivering the *SMA1* transgene—effectively restoring motor functions in SMA patients [157, 158]. Further studies are needed in specifically exploring the differences in transduction efficiency of therapeutics between intravenous *vs* intracranial/intrathecal injections in model organisms.

Although AAVs have traditionally been the main vector used to deliver CRISPR-Cas9 constructs, the relatively small packaging size (~4.5 kb) makes it so that researchers must deliver two separate vectors containing the CRISPR-Cas9 construct (~4.2 kb) and gRNA separately. In particular with the CRISPRa approach, the transcriptional activators that are fused to the dCas9 enzyme will increase the length of the construct, such that a combination of sgRNA and dCas9-transcriptional activators will be too large to load onto a single AAV vector. This is less desirable in a clinical setting as it would necessitate the production of two AAV’s, adding significant cost to the development of the therapeutic.

Given that the traditional CRISPR-Cas9 system was derived from the *Streptococcus pyogenes* bacteria (SpCas9), recent techniques have aimed to overcome this size limitation by investigating the efficacy of other smaller Cas9 orthologues. One such example involves utilizing a smaller Cas9 ortholog from *Staphylococcus aureus* (SaCas) (3.15 kb), which is ~1 kb shorter than the SpCas9, so that the CRISPR-Cas9 construct and gRNA can fit within a single AAV vector [159, 160]. This technique was successful in restoring the expression of the *UBE3A* sense gene via NAT degradation in a mouse model of AS, as well as rescuing disease phenotypes in a DMD mouse model [58, 161]. Similarly, Cas9 from *Campylobacter jejuni* (CjCas9) (2.95 kb) has been used in single-AAV vectors to successfully decrease the choroidal neovascularization phenotype in a mouse model of age-related macular degeneration (AMD) [162]. Further experiments should investigate the efficacy of CjCas9 in rescuing phenotypes associated with NDDs.

Since the BBB also prevents most small-molecule drugs in addition to the previously mentioned viral vectors from being able to enter the CNS, the recent development of AAV9 variants have been a potential key in solving this issue. Variants such as rAAV-PHP.B and a second generation rAAV-PHP.eB contain an engineered capsid—contributing to an unprecedented efficiency in crossing the BBB upon intravenous injection, along with high diffusion capacity into both neurons and glia of mice [163–166]. The high BBB-crossing efficiency of rAAV-PHP.B and rAAV-PHP.eB is due to their interactions with the LY6A (also known as SCA-1) protein expressed on the brain microvascular endothelial cells (BMVEC) of the mouse BBB [167]. Unfortunately, LY6A is not expressed in primates—providing a major translational obstacle for human BBB permeation. In light of these translational obstacles for rAAV-PHP.eB and rAAV-PHP.B, one recent study produced AAV.CPP16 by inserting cell-penetrating peptides (CCPs) into the AAV9 capsid between Q588 and A589 amino acids [168]. Compared to unmodified AAV9, intravenous injection of AAV.CPP16 in mice showed substantially greater BBB-crossing efficiency, greater transduction efficiency, and higher neuron specificity within multiple mouse strains and cynomolgus macaques. Although the AAV.CPP16 vector shows promise for the future of CNS-targeting therapeutics, further studies are needed to confirm successful rescue in disease models.

Neurotransmitter-derived lipidoids (NT-lipidoids) have recently become a new promising delivery vector, especially for the CNS. One study recently demonstrated that a NT-lipidoid vector successfully introduced a Tau-targeting ASO, small molecule drug, and a fusion protein into the brain of mice via intravenous injection [169]. Similarly, nanoparticles can potentially be used to deliver therapeutics to the brain in a two-step targeting strategy that exploits the high impermeability of the BBB to selectively retain ligand labels on the surface of brain endothelium [170]. Evidence from other studies have shown that ASOs, mRNA, CRISPR-Cas9 constructs, and small molecule drugs can be delivered using a lipid nanoparticle vectors [171–174]. Therefore, developing better vectors for brain- and neuron-specific targeting could further advance NDD therapeutic strategies.

## Discussion

This review has investigated three main targets of intervention for NDDs across the central dogma, specifically at the: DNA, mRNA, and protein levels. While there has been much success within in vitro and animal models at the DNA and protein levels, there has yet to be successful translation of these therapeutics in human clinical trials. The various therapeutic techniques discussed within this review that have either been in or successfully completed clinical trials have been summarized in Table 2.

**Table 2:** Summary of therapeutics discussed in the review.

Therapeutic	Type	Target	Disease	Current Status	Source [PMID/NCT]
DNA-Level					
Luxturna	Transgene Delivery	*RPE65* Gene	Retinal Dystrophy	FDA Approved	PMID: 30711576
EDIT-101	CRISPR Genome Editing	*CEP290* Gene	Leber Congenital Amaurosis (LCA10)	Currently in Phase 1/2 Trials	NCT: NCT03872479
RNA-Level					
Spinraza	Exon-Inclusion ASO	*SMN2* mRNA	Spinal Muscular Dystrophy (SMA)	FDA Approved	PMID: 28400976
Drisapersen	Exon-Skipping ASO	*DMD* mRNA	Duchenne Muscular Dystrophy(DMD)	Failed in Phase 3 Clinical Trials	PMID: 29203355
Eteplirsen	Exon-Skipping ASO	*DMD* mRNA	Duchenne Muscular Dystrophy(DMD)	FDA Approved	PMID: 29203355
Milasen	Splice-Switching ASO	*CLN7* mRNA	Batten’s Disease	FDA Approved	PMID: 31597037
GTX-102	NAT-targetingASO	*UBE3A-AS* mRNA	Angelman Syndrome(AS)	Currently in Phase 1/2 Trials	NCT: NCT04259281
ION582	NAT-targeting ASO	*UBE3A-AS* mRNA	Angelman Syndrome (AS)	Currently in Phase 1/2 Trials	NCT: NCT05127226
RO7248824	NAT-targeting ASO	*UBE3A-AS* mRNA	Angelman Syndrome (AS)	Currently in Phase 1/2 Trials	NCT: NCT04428281
Protein-Level					
Arbaclofen	Small Molecule Drug	GABA-B Receptor	Fragile X Syndrome (FXS)	Failed in Phase 3 Clinical Trials	PMID: 28616094
Mavoglurant	Small Molecule Drug	mGluR5	Fragile X Syndrome (FXS)	Failed in Preclinical Trials	PMID: 26764156
Everolimus	Small Molecule Drug	mTOR Complex	Autism (ASD) and Tuberous Sclerosis Complex (TSC)	Failed in Phase 3 Clinical Trials	PMID: 31217257

PMID denotes the identification number for sources located in PubMed. NCT denotes the ClinicalTrials.gov Identifier for the respective clinical trial.

*NAT -* Natural Antisense Transcript, *ASO -* antisense oligonucleotide.

Efforts to treat gene haploinsufficiency will necessitate careful consideration of cell type specificity, tissue specificity, and modulating the expression of the correct splicing isoforms—something that will vary across tissues and developmental stages [175]. Alternative splicing plays important role in NDDs, and ASD risk genes have differentially expressed isoforms (DEI) during every stage of prenatal development [176–178]. Furthermore, DEIs impact pathways involved in dendrite development, synapse organization, and neuronal projection, the processes that are dysregulated in ASD [12, 177]. One study found that in *NRXN1*^*+/-*^ human induced pluripotent stem cell (hiPSC)-derived neurons, there was a significant increase in expression of novel *NRXNα* isoforms, coupled with a decrease in the wild-type *NRXNα* isoform [179]. This dysregulation in *NRXNα* isoform balance resulted in a reduction of neuronal activity and disruptions in neuronal maturation. Therefore, during the therapeutic development process, it is imperative to consider patient age, cell-type specific isoforms, alternative start sites, and alternative promoters for target genes. The strength of the promoter in transgene therapy or modulation of the CRISPRa transcriptional activators will play a key role in determining the appropriate treatment and avoiding unpredictable or undesired treatment consequences.

With the recent efforts of shifting focus to the therapeutic rescue of genes in NDDs, it may be possible that that the next decade will yield translational success. This success is dependent on further optimization of techniques still in their infancy such as CRISPRa or uORF-based/NMD-inhibiting ASOs. Furthermore, therapeutic mechanisms such as alternative splicing ASOs that have FDA approval for non-NDD pathologies such as Spinraza (SMA) and Eteplirsen (DMD) may provide a strong basis for further studies using these ASOs for genes with splice mutations implicated in NDDs. As evidenced by the mTOR inhibition therapeutics for TSC, the time point of therapeutic administration is also an important consideration for successful translation into clinic.

When it comes to NDDs, it will be of paramount importance to diagnose as early as possible in order to administer the therapeutics during the critical brain developmental periods. With this in mind, studies have shown that the late mid-fetal to early postnatal period is a critical window for neurodevelopment, in which many ASD risk genes are expressed [180–182]. An example of such can be observed in a study where Cre-dependent activation of *Ube3a* in *Ube3a*^*Stop/p+*^ embryonic mice restored all AS-associated motor, behavioral, and neurological deficits, whereas *Ube3a* reactivation in postnatal mice demonstrated diminishing efficacy with age for motor coordination rescue [183]. With the developing state of CNS-targeting therapeutic delivery strategies and the potential complications involved with delivering prenatal therapeutics, NDD’s are likely to be the most effectively treated during the early postnatal period. It is important to note that these time points will not be universally applicable for all genes and all phenotypes. Other studies have demonstrated successful rescue of neurological and electrophysiological deficits in adult *Ube3a*^*Stop/p+*^ mice after reactivation of the *Ube3a* gene and rescue of neurological defects in adult *Mecp2*^*lox-Stop/y*^ mice after reactivation of the *Mecp2* gene [183–185]. Similarly, the previously discussed *Scn2a* CRISPRa study successfully rescued electrophysiological deficits in adolescent *Scn2a* haploinsufficient mice [78]. This heterogeneity in phenotypic rescue across different developmental time points suggests that periods of therapeutic intervention will need to be assessed on a gene-to-gene basis.

With mouse models not being able to accurately recapitulate human neurodevelopmental periods, another translational step could be developing models of haploinsufficiency within NHPs—something that has already been established for the SHANK3 and MECP2 genes within cynomolgus macaques [35, 36]. The common marmoset (*Callithrix jacchus*) is another potential model organism for human NDD’s, with studies showing that the gene expression and gene distribution patterns within the brains of humans and marmosets were more similar than that of humans and mice [186]. In addition to this, another study was able to successfully develop a transgenic marmoset model of Huntington’s disease (HD) [187]. These marmosets displayed dystonia and chorea—forms of involuntary movement that are physiological phenotypes of HD. Given these results, it may be valuable to pursue transgenic NHP models of NDD’s, with studies evaluating the efficacy of therapeutic treatments across different neurodevelopmental time points.

Another obstacle to clinical translation involves optimization of delivery methods for the therapeutics. When considering the use of viral vectors in NDDs, some important considerations include balancing tissue-specificity, immunogenicity, packaging limits, and ability to penetrate the BBB. With the advent of technologies such as lipid-based vectors, it may be possible to overcome the obstacles associated with the viral vectors for the development of therapeutics. However, since the lipid-based vectors are still in their infancy, further studies are needed to clearly compare both efficacy and safety between viral and lipid-based vectors.
