# Correction to: Therapeutic strategies for autism: targeting three levels of the central dogma of molecular biology

**DOI:** 10.1038/s41398-023-02386-6

**Published:** 2023-03-07

**Authors:** Derek Hong, Lilia M. Iakoucheva

**Affiliations:** 1 Department of Psychiatry, University of California San Diego, La Jolla, CA USA; 2 Division of Biological Sciences, University of California San Diego, La Jolla, CA USA; 3 Institute for Genomic Medicine, University of California San Diego, La Jolla, CA USA

**Keywords:** Molecular neuroscience, Personalized medicine, Autism spectrum disorders

Correction to: *Translational Psychiatry* 10.1038/s41398-023-02356-y, published 16 February 2023

The original version of this article contained an error in the text. The correct sentence should be: “Similarly, nanoparticles can potentially be used to deliver therapeutics to the brain in a two-step targeting strategy that exploits the high impermeability of the BBB to selectively retain ligand labels on the surface of brain endothelium [170].” The original article has been corrected.

